# On the Diagnosis of Tape-Worm

**Published:** 1851-02

**Authors:** 


*On the Diagnosis of Tape-worm, from the character of the Nervous Symptoms induced by its presence.*—M. Legendre has published an interesting memoir, upon the nervous symptoms produced by tape-worm, in the *Archives Générales de Médecine* for June, 1850. He places the facts of this department of diagnosis in a very clear point of view; and, on this account, we think it right to call the special attention of our readers to the following brief abstract of his paper. When anomalous nervous symptoms perplex the physician, it is always right to compare them with those which are known to be caused by tape-worm; and if any room for suspicion of its presence exist, it is always imperative to administer a vermifuge. We say *always*, because the experiment is at least harmless, as the perfect safety, as well as the general success of the kousso has now been fully established on unimpeachable authority. We would in all cases, prefer this medicine, or the oil of the male fern, to the bark of the root of the pomegranate, which is considered by M. Legendre to be the best. The latter is likewise, undoubtedly, a most valuable drug for dislodging the tænia; but it is not quite equal to the kousso, or the male fern. The following is the substance of M. Legendre's paper:

Thirty-three fully reported cases are carefully analyzed.

*Disorders of the cerebro-spinal nervous system* occurred most frequently; viz., in twenty of the thirty-three cases. They were in twelve cases, of a convulsive character, occurring several times, or oftener (being eight times epileptiform, and four times hysterical). In eight of the cases the convulsive movements were partial, being confined to the face, or to a single limb. In fourteen of the thirty-three cases, the convulsions were succeeded by vertigo and cephalalgia.

*Swooning*, complete or incomplete, was noted in about a fifth of the cases; viz., seven times.

*Disturbed vision* was noted in six of the thirty-three cases. This consisted in double vision or in the presence of flocculi, of *muscæ volitantes*, and of luminous flashes. One patient is mentioned as having had periodical blindness.

*Buzzing in the ears* is only mentioned as having occurred in three of the cases.

*A pricking or gnawing sensation at the epigastrium* existed in fourteen of the thirty-three patients. The symptoms now enumerated rarely existed singly in one patient, and two or three of them were generally present in the same individual.

Whenever one or more of the above symptoms are observed and no other explanation can be given, an investigation should be made to determine whether they may not depend upon tape-worm. If the physician discover that the patient have a gnawing at the stomach, capricious, or insatiable appetite, abdominal pain, a feeling of general prostration, or itching at the entrance of the nostrils or anus, there can be little doubt of the presence of the parasite. M. Legendre believes that the best time for giving vermifuge is when the symptoms are most urgent, or when some parts have been spontaneously voided. He says that Wawruch, of Vienna, in an analysis of two hundred and six cases, attributes such occurrences to lunar influence; asserting that they occur most frequently at the full moon, or during its decline, and that it is, likewise, at these times that the patient suffers from an exacerbation of the symptoms. It was not till M. Legendre had finished his researches that he became acquainted with the analysis of M. Wawruch's paper, published several years ago, and which, from its value, we subjoin.—*London Journ. Med.* Nov. 1850.

—

*Wawruch on the Causes and Symptoms of Tape-worm.*—The following is an extract from an abstract, contained in the *Gazette Médicale* for 2d October, 1841, of a paper published by Professor Wawruch in the *Medicinische Jahrbucher der Œsterreichischen Staaten*:

During the period of twenty years, Dr. Wawruch treated 206 persons affected with tænia; 71 men, and 135 women. The oldest person was aged fifty-four; the youngest, three and a half years. Twenty-two were under fifteen years of age; and among these were six girls who had not menstruated. Most of the patients were from fifteen to forty years old. The patients belonged to the middle and lower classes; they nearly all inhabited the district lying along the Danube, or lived in low and damp dwellings. They followed very different occupations, and their mode of life was various. It is remarkable that the same conditions which give rise to scurvy in Vienna also produce tape-worm. The articles of food which seemed chiefly to engender tape-worm were bad bread, meals of milk, butter, cheese, potatoes, pork and mutton, and bad water. The diseases which preceded the formation of tænia were gastric and cutaneous affections, but especially gastric and intermittent fevers. There were observed forty-three cases of intermittent fever, twenty of gastric fever, sixteen of typhoid fever, ten of ringworm and herpes, forty-two of itch, eight of scarlatina, thirteen of measles, and two of chronic urticaria. Scurvy, syphilis, chlorosis, and other diseases which affect digestion, were also observed to precede tænia; but, in general, few of the individuals affected with tape-worm had not had lumbrici when young. The influence of hereditary predisposition is very doubtful. Dr. Wawruch only saw two instances; in one, a mother and daughter, in another, a father and son had tænia. Irregularity of the menstrual function seemed common; thus, thirteen females had only menstruated at the age of 16 years, twelve at 17, nine at 18, seven at 19, and one at 20. The duration of the disease was from a few months to ten, twelve, fifteen, twenty, twenty-five, and, in one case, even to thirty-five years. Of the 206 patients, three only, who were foreigners, had *bothriocephalus;* the others had *tænia solium.*

*Symptoms.*—The symptoms of tænia are very various; the following are stated by Dr. Wawruch to be the most constant:

1. Dull pain in the frontal region, vertigo, noises in the ears; and, in some a kind of idiosyncrasy for music.

2. Eyes dull, surrounded by a blue ring; œdema of the upper eyelid; pupils dilated; frequent involuntary action of the muscles of the eye; various aberrations of sight, as amblyopia, diplopia, or muscæ volitantes. One patient complained of a periodical blindness during the day.

3. Frequent change in the color of the face; lips leadcolored; a peculiar look about the mouth and nose; the appearance of general cachexia is not constant, for the patients often preserve a flourishing aspect for some time: many of them say they have become thinner.

4. Anorexia, alternating with such a voracious appetite that the patients faint if they are not supplied quickly enough with food; desire for certain articles, as carrots, milk, wine, bread, etc.

5. Feted breath, clayey taste, loaded tongue, salivation, nausea, and even vomiting of liquid, as clear as water, in the morning.

6. Itching of the nose and anus, and the vagina in females; grinding of the teeth, especially during sleep; accumulation of a clear fluid in the mouth; transient heat, langor, palpitation. The sensation of a cold hand pressing on the heart (considered as a pathognomic symptom by Reinlein) has never been observed.

7. Abdomen tumefied; borborygmi; a sensation of suction, constriction, and pricking, around the umbilicus, and undulatory movements, as of a foreign body in the intestines—especially in the morning; cessation of these symptoms after taking farinacious food, hot bread, or *café au lait;* frequently diarrhea, alternating with constipation.

8. When the disease has continued for some time, and the patients are of an irritable temperament, there often arise a train of nervous symptoms, as melancholia, syncope, aberrations of the senses, disturbed sleep, frightful dreams, partial and general convulsions, chorea, epilepsy, aphonia, and loss of speech.

9. The most certain pathognomonic symptom is the evacuation of one or more portions of tape-worm, either spontaneously, or after certain severe illnesses—as scarlatina, typhoid fever, pneumonia, etc.; or after the use of certain articles of food, as garlic, horseradish, cucumber, etc.; or after the administration of anthelmintic medicines. The portions are sometimes evacuated at indeterminate periods, but generally at new moon, or at the decrease of the moon; and at these times, also, the other symptoms are increased in severity.—*Lond. Jour. Med.*, Nov. 1850.

